# Overactivation of GnRH neurons is sufficient to trigger polycystic ovary syndrome-like traits in female mice

**DOI:** 10.1016/j.ebiom.2023.104850

**Published:** 2023-10-27

**Authors:** Mauro S.B. Silva, Laurine Decoster, Gaspard Delpouve, Tori Lhomme, Gaetan Ternier, Vincent Prevot, Paolo Giacobini

**Affiliations:** a Laboratory of Development and Plasticity of the Neuroendocrine Brain, FHU 1000 Days for Health, School of Medicine, Lille, France; b Univ. Lille, Inserm, CHU Lille, Lille Neuroscience & Cognition, UMR-S 1172, Lille, France

**Keywords:** PCOS, GnRH, Ovarian dysfunction, Luteinizing hormone, Hyperandrogenism

## Abstract

**Background:**

Polycystic ovary syndrome (PCOS) is the most common endocrine disorder leading to anovulatory infertility. Abnormalities in the central neuroendocrine system governed by gonadotropin-releasing hormone (GnRH) neurons might be related to ovarian dysfunction in PCOS, although the link in this disordered brain-to-ovary communication remains unclear. Here, we manipulated GnRH neurons using chemogenetics in adult female mice to unveil whether chronic overaction of these neurons would trigger PCOS-like hormonal and reproductive impairments.

**Methods:**

We used adult *Gnrh1*^*cre*^ female mice to selectively target and express the designer receptors exclusively activated by designer drugs (DREADD)-based chemogenetic tool hM3D(Gq) in hypophysiotropic GnRH neurons. Chronic chemogenetic activation protocol was carried out with clozapine N-oxide (CNO) i.p. injections every 48 h over a month. We evaluated the reproductive and hormonal profile before, during, and two months after chemogenetic manipulations.

**Findings:**

We discovered that the overactivation of GnRH neurons was sufficient to disrupt reproductive cycles, promote hyperandrogenism, and induce ovarian dysfunction. These PCOS features were detected with a long-lasting neuroendocrine dysfunction through abnormally high luteinizing hormone (LH) pulse secretion. Additionally, the GnRH-R blockade prevented the establishment of long-term neuroendocrine dysfunction and androgen excess in these animals.

**Interpretation:**

Taken together, our results show that hyperactivity of hypothalamic GnRH neurons is a major driver of reproductive and hormonal impairments in PCOS and suggest that antagonizing the aberrant GnRH signaling could be an efficient therapeutic venue for the treatment of PCOS.

**Funding:**

10.13039/501100000781European Research Council (ERC) under the European Union’s Horizon 2020 research and innovation program (grant agreement n◦ 725149).

**Research in context**

**Evidence before this study**

PCOS is highly prevalent among women of reproductive age affecting fertility, hormonal balance, and general quality of life. Although the disease’s etiology remains mostly cryptic, abnormally high secretion of luteinizing hormone (LH) is common among PCOS patients hinting that gonadotropin-releasing hormone (GnRH) neurons are under a hyperactive state. Thus, we hypothesized that the overactivation of GnRH neurons is a primary trigger for the manifestation of PCOS symptoms using adult female mice as a pre-clinical model.

**Added value of this study**

Here, we innovatively used chemogenetic manipulations to chronically activate GnRH neurons to promote neuroendocrine dysfunction in female mice as observed in PCOS. We found that chronic overactivation of GnRH neurons recapitulates all diagnostic features and hormonal imbalance of PCOS in this murine model and that GnRH signaling is required to relay the central dysfunction to the disorder’s hormonal imbalance and ovarian disorder.

**Implications of all the available evidence**

Our findings demonstrate that an initial central trigger leading to enhanced GnRH neuron activity over adult life is sufficient to promote PCOS-like reproductive and hormonal disturbances. These results emphasize the importance of understanding central mechanisms affecting GnRH release and signaling in the etiology of PCOS.

## Introduction

Polycystic ovary syndrome (PCOS) is a highly prevalent endocrine disorder affecting about 10% of women of reproductive age globally.^1^^,^^2^ According to more recent international guidelines,^3^ PCOS is diagnosed upon the identification of two out of its three main features: hyperandrogenism (androgen excess), menstrual irregularities, and the presence of polycystic-like ovarian morphology. The disease is also strongly linked to infertility, metabolic syndrome, and mental health-related burden conditions with unclear pathogenic roots,^4^, ^5^, ^6^ bearing challenges for women’s health management and high costs for its available treatments.^7^

Since its initial description from Stein and Leventhal’s work,^8^ a long-standing focus has been granted to PCOS ovaries, from their functional determination of androgen production, morphological features, and association to metabolic comorbidities.^3^^,^^9^ Despite the assured relevance of ovarian dysfunction, an investigative shift in the field has revealed the role of disturbances in the female brain with neuroendocrine and reproductive consequences for PCOS women. Clinical studies report that most PCOS patients exhibit higher luteinizing hormone (LH) pulsatile secretion, which hints at central alterations affecting gonadotropin-releasing hormone (GnRH) neurons^10^, ^11^, ^12^, ^13^, ^14^, ^15^, ^16^, ^17^, ^18^, ^19^ and recent investigations performed by us also highlighted the brain’s role in the neuroendocrine dysfunction of PCOS both in rodents and humans.^20^ GnRH neurons regulate fertility through the pulsatile release of the GnRH peptide to the pituitary gland coordinating LH and follicle-stimulating (FSH) hormone secretion.^21^ Both gonadotropins operate at the ovarian level to drive gonadal development, steroidogenesis, and ovulation in healthy females. Importantly, GnRH and LH have a one-to-one relationship, which enables the indirect assessment of GnRH function through its proxy LH levels in the peripheral circulation.^22^^,^^23^ Reflecting back on PCOS, nearly 70–75% of these patients present increased LH pulse frequency, regardless of their body mass index (BMI) and more than 90% of PCOS patients have a persistently high LH to FSH ratio,^11^^,^^24^, ^25^, ^26^ which suggests the presence of an increased GnRH pulse frequency in women with PCOS. Elevated GnRH/LH release may negatively impact ovarian follicular development,^27^ favour androgen hypersecretion,^28^^,^^29^ and impair ovulation^30^^,^^31^ in PCOS. Thus, chronically enhanced GnRH neuronal activity might be a key primary factor leading to reproductive impairments in PCOS.

Clinical and pre-clinical studies indicate that high GnRH/LH output during pubertal development might initiate altered endocrine interactions between the brain and ovaries promoting hyperandrogenemia, which acts back to the neuroendocrine circuits to maintain altered GnRH/LH secretion over reproductive life.^18^^,^^27^^,^^32^, ^33^, ^34^ Although this “neuroendocrine primary trigger” scenario is proposed to substantially contribute to the PCOS etiology, whether an initial trigger from abnormally high GnRH neuron activity and secretion might be sufficient to drive PCOS symptomology remains unknown. Here, we focused on determining whether chronic overactivation of GnRH neurons promotes the appearance and maintenance of the major PCOS-like traits over time. To this end, we used chemogenetic manipulations to chronically induce high GnRH neuron activity in adult mice interrogating whether this initial central trigger is sufficient to initiate a vicious cycle of neuroendocrine disturbances and reproductive impairments that mimic the adult PCOS-like pathology. We uncovered that enhancing GnRH neuron activity promotes long-lasting neuroendocrine dysfunction, impairments in reproductive cycles, hyperandrogenemia, and ovarian dysfunction. Notably, reproductive, and endocrine derangements remained even two months after the withdrawal of the chemogenetic agonist treatment, and their appearance was prevented by antagonizing GnRH receptor signaling at the time of high GnRH neuron activity. Our results highlight GnRH dysfunction in the female brain as a major driving factor of PCOS and point to this new PCOS mouse model as a valuable tool for future studies aimed at understanding the pathophysiology of PCOS.

## Methods

### Animals

Mice were grouped-housed in our animal breeding facility equipped with pathogen-free rooms in closed ventilated cages under temperature control at 21–22 °C, 12:00 h light: dark cycle control, and *ad libitum* access to food and water. A standard diet (9.5 mm Pelleted RM3, Special Diets Services; Competence Centre for Lab Animal Science of SAFE®; France) was given to all mice during the experimental period. All experimental procedures commenced when mice were 8–10 weeks old. Wild-type and transgenic mouse lines are described in the SI Appendix. Mice were randomly assigned to groups of five animals per cage at the time of weaning with litters within the same treatment allocation group and mixed together (pseudo-random mixing based on weight) to minimize any potential bias. No data were excluded from analyses.

### Prenatal anti-Müllerian hormone (PAMH) mouse model

PAMH mice were generated according to previous studies.^35^ Pregnant adult (3–4 months) female mice were injected daily intraperitoneally (i.p.) at gestational day (GD) 16.5, 17.5, and 18.5 with either 200 μL of 0.01 M phosphate-buffered saline (PBS) only (control group) or 0.12 mg/kg/day human recombinant AMHC (R&D Systems, rhMIS 1737-MS-10) diluted in PBS (PAMH group). Mouse pregnancy was timed considering the detection of vaginal plugs as GD 0.5.

### Assessment of reproductive status and blood sampling

Reproductive cycles were followed and characterized according to well-described guidelines of the mouse estrous cycle elsewhere.^36^ Briefly, vaginal smears were collected using 10 μL of sterile saline, transferred to glass slides, and evaluated during the morning period at 8:00–9:00 h (room lights were on at 7:00 h). Vaginal cytology was performed with freshly collected samples using an inverted microscope with maximum microscope condenser distance allowing a better contrast for proper visualization. Estrous cyclicity was followed during 16 consecutive days at each period of evaluation: before, during, and after CNO treatment. Blood sampling for LH and testosterone levels were collected when mice were in diestrus. For LH-level profile studies, mice were trained to interact with the investigator and allow restraint of the tail for at least one week before the commencement of each set of experiments. A 4 μL of the whole blood sample from the tail tip was taken every 6 min (LH profile) for 2 h or 12 min (CNO-mediated LH release) for 72 min. Samples were collected into Eppendorf tubes pre-loaded with 50 μL of 0.1 M phosphate-buffered saline (PBS)-0.05% Tween (pH = 7.4), thoroughly mixed, and snap-frozen with dry ice and stored at −80 °C. Plasma testosterone samples were collected from the tail tip at a single point for each treatment CNO period (before, during, and after CNO treatment) and following the Cetrorelix experimental set. Samples were kept in ice for 1 h followed by centrifugation at 12,000 rpm for 15 min at 4 °C. Plasma supernatant was transferred to sterile 200-μL Eppendorf tubes and stored at −80 °C.

### Hormone measurements and analysis

For this study, we used an ultrasensitive ELISA method to measure circulating LH levels in the whole blood in mice as validated previously to assess pulsatile LH release in female mice over the estrous cycle.^37^^,^^38^ Briefly, this ELISA uses a 96-well high-affinity binding microplate (Corning) coated with 50 μL of primary capture antibody (bovine LHβ subunit, 518B7; L. Sibley; University of California, UC Davis) at a dilution of 1:1000. The mouse LH-RP reference used for this assay was provided by Dr. Albert F. Parlow (National Hormone and Pituitary Program, Torrance, California, USA) and used to generate a standard curve with a two-fold serial dilution from a start standard of 4 ng/mL to 0.0019 ng/mL LH-RP reference diluted in 0.2% bovine serum albumin (BSA)-0.1 M PBS-0.05% Tween solution. Whole blood samples were transferred to the coated as singlets for each point of sampling and incubated for 2 h under agitation at room temperature. A rabbit LH antiserum primary antibody (AFP240580Rb; NIDDK-NHPP) was used at 1:10,000 dilution and a secondary horseradish peroxidase-conjugated antibody (goat anti-rabbit; Vector Laboratories, PI-1000) was used at 1:10,000 dilution. We used 100 μL of 1-Step Ultra TMB-Elisa Substrate Solution (ThermoFisher Scientific, cat. #34028) for the final revelation step followed by a stop solution with 50 μL of 3 M HCl per well. The assay sensitivity of this LH ELISA was 0.04 ng/mL and intra-assay coefficient of variation was 3.9% and the inter-assay coefficient of variation was 8.3%. LH pulse analysis was analysed based on previous studies elsewhere.^38^, ^39^, ^40^ Briefly, mean LH levels were considered as the average of all measured values within the 2-h blood sampling protocol. Following the visualization LH pulse profile using Prism 9.0.0 software (GraphPad Software, Dotmatics; San Diego, CA, USA), LH pulse peaks were determined as a single point with a value of more than 10% of the preceding nadir point. Next, LH pulse frequency was determined by counting the number of identified pulses per hour. The average time distance (in min) between each LH pulse peak was used to calculate the LH pulse interval. The LH pulse amplitude was calculated considering the peak of each LH pulse minus its preceding zenith values followed by a final average calculation of all amplitudes within the 2-h blood sampling protocol. Basal LH level analysis considered the average of all LH nadir and non-peak values. The area under the curve (AUC) analysis is considered the total area summed over the 2-h blood sampling protocol using Prism 9.0.0 software and is interpreted as an integrated response to putative GnRH-mediated LH release over time as previously shown elsewhere in PCOS clinical^13^ and pre-clinical^41^ studies. Plasma testosterone levels were measured in duplicates using a commercial mouse ELISA kit following the manufacturer’s instructions (Demeditec Diagnostics, GmbH, DEV9911). The assay sensitivity of this mouse testosterone ELISA was 0.066 ng/mL, and the intra-assay coefficient of variation was 8.9%. Quality controls from previous studies in our laboratory were included in this assay and guaranteed replicability standards. Plasma AMH levels were measured using a commercial rat and mouse ELISA kit following the manufacturer’s instructions (AnshLabs®, Cat. No. AL-113). The assay sensitivity of this ELISA was 0.011 ng/mL as reported by the manufacturer, and the intra-assay coefficient of variation was 5.2% as analysed in singlicates. Quality controls were also provided by the manufacturer.

### Surgical procedures: viral transfection and stereotaxic injections

Mice (8–10 weeks old) were anesthetized with 2% isoflurane and placed in a stereotaxic apparatus (Stoelting Co., Wood Dale, IL) with head and nose fixed. All experiments were performed with sterile instruments and aseptic conditions and mice received a pre-surgical s.c. injection of 5 mg/kg carprofen as systemic analgesic. Stereotaxic coordinates were chosen according to the adult mouse brain atlas^42^ to target the ARN and ME region where hypophyseal GnRH neurons project to as anterior-posterior = −1.8 mm, medial-lateral = ±0.25 mm, and dorsal-ventral = −5.9 mm to target. Bilateral injections were performed using a 2 μL Neuros Hamilton syringe (#7002 series; Hamilton©, Reno, Nevada, USA) filled with 300 nL of AAV9-hSyn-DIO-hM3D(Gq)-mCherry (Addgene viral prep #44361-AAV9; 0.48 × 10^13^ GC/mL; a gift from Professor Bryan Roth, UNC—Chappel Hill, USA) and injected in each side at a rate of 50 nL/min. Syringes were left *in situ* for 5 min before and 10 min after the viral injection.

### Acute and chronic *in vivo* chemogenetic activation protocol

Acute *in vivo* chemogenetic activation protocol used diestrous female mice i.p. injected with either saline or three different doses of CNO (0.2, 1.0, and 5.0 mg/kg; Clozapine N-oxide dihydrochloride; Tocris, Cat. No. 6329). Mice were pre-handled for a week to endure tail-tip blood sampling and received two i.p. sham injections before starting these experiments. Chronic *in vivo* chemogenetic activation protocol was performed using 1 mg/kg CNO i.p. administered every 48 h and tail-tip blood samples were collected only when mice did not receive this i.p. CNO injection. Two groups were generated for this protocol: one in which blood samples were used to measure pulsatile LH secretion and a second one to measure plasma testosterone levels. Each group consisted of two experimental rounds; thus, the present results were replicated and collected in duplicate. A particular exception was made for the GnRH signaling blockade experiments with Cetrorelix in which blood samples were collected from the same group of mice to measure both LH and testosterone levels.

### Cetrorelix treatment

The GnRH antagonist Cetrorelix (Cetrorelix acetate; Sigma-Aldrich #C5249) was co-administered with 1 mg/kg CNO and both were diluted in sterile saline. The dose of 0.5 mg/kg of Cetrorelix was chosen according to previous work in our laboratory.^35^

### Immunohistochemistry in brain sections: tissue preparation, immunostaining, image acquisition, and quantification

Female mice in diestrus were anesthetized with i.p. injections of 100 mg/kg of ketamine-HCl and 10 mg/kg xylazine-HCl and perfused transcardially with 10 mL of cold saline, followed by 30 mL of cold 4% paraformaldehyde (PFA) in 0.1 M phosphate buffer (PB) (4% PFA/0.01 M PB; pH 7.6). Brains were postfixed in the same fixative solution overnight at 4 °C, transferred into a Tris-buffered saline (TBS) TBS-30% sucrose solution, and kept at 4 °C for one week. Fixed brain tissue was cut into four series of 35-μm-thick coronal sections using a freezing microtome. Free-floating coronal sections were chosen and analysed using the mouse brain atlas as a reference.^42^ Sections were rinsed using 0.05 M TBS (pH = 7.6) followed by incubation in a blocking solution (0.05 M TBS; 0.3% Triton-X100; 0.25% Bovine-serum-albumin; 5% Normal Donkey Serum, NDS) for 1 h at room temperature (RT). Next, brain sections were transferred to an incubation solution (0.05 M TBS; 0.3% Triton-X100; 0.25% Bovine-serum-albumin) with primary antibodies and 2% NDS for 48 h at 4 °C. Primary antibodies were: chicken anti-GFP (chicken; dilution 1:1000; Aves-Lab GFP-1010) and rabbit anti-RFP (dilution 1:1000; Rockland Immunochemicals, Item No. 600-401-379). Next, sections were washed with 0.05 M TBS and incubated with the secondary antibodies Alexa Fluor 488 (1:500) and Alexa Fluor 568 (1:500) (Thermo Fisher Scientific). Sections were then coverslipped with Fluoromount-GTM with DAPI (Invitrogen, REF: 00-4959-52) and stored at 4 °C. Two representative sections were selected from each mouse and GnRH cell counts were performed on one out of four collection series. Results were averaged and multiplied by four to correct the number of series. Brain sections were imaged using an Axio Imager Z2 ApoTome microscope equipped with a motorized stage (Zeiss, Germany) and an ORCA-Flash 4.0 V2 camera (Hamamatsu, Japan) driven by the Zen imaging software (Zeiss). Z-stack images were taken using a 1-μm step through the whole thickness of each representative section. ImageJ (National Institutes of Health, Bethesda, MD) and Photoshop CS5 (Adobe Systems, San Jose, CA) were used to process, quantify, adjust, and merge the photomontages.

### Brain slice preparation for electrophysiological recordings and patch-clamp recording

Electrophysiological recordings were performed on living brain slices from 8 to 12-week-old mice. Mice were put under isoflurane anesthesia and killed by decapitation. The brain was dissected and rapidly placed in ice-cold aCSF containing: 120 mM NaCl, 3.2 mM KCl, 1 mM NaH_2_PO_4_, 26 mM NaHCO_3_, 1 mM MgCl_2_, 2 mM CaCl_2_, 10 mM glucose (300 mOsm, pH 7.4) and bubbled with 95% O_2_ to 5% CO_2_. 200 μm coronal slices containing the rostral preoptic area were cut using a VT1200 vibratome (Leica). Slices were incubated at 34 °C in oxygenated aCSF for a recovery period of 1 h and then placed at room temperature until patch-clamp recording. Individual brain slices were placed in a submerged recording chamber (Warner Instruments) and continuously perfused at a rate of 3 mL/min with oxygenated aCSF maintained at 32.8 °C by a heater controller (TC-344C-Warner Instrument). GnRH neurons were visualized under ×10 and ×40 magnification using an upright fluorescence microscope with infrared differential interference contrast (DM-LFSA, Leica) and an ORCA-Frash4.0 digital CMOS camera (Hamamatsu). Recording pipettes were pulled from borosilicate glass capillaries (1.5 mm outer diameter, 1.12 mm inner diameter; World Precision Instruments) using a P1000 Flaming Brown puller (Sutter Instruments) and had a resistance of 7–9 MΩ when filled with an internal solution containing 140 mM K-gluconate, 10 mM KCl, 1 mM EGTA, 2 mM Na_2_-ATP, and 10 mM HEPES, pH 7.3, with KOH. Whole-cell patch-clamp recordings were performed in current-clamp mode using a Multiclamp700B Amplifier, digitized with the Digidata 1322A interface, and acquired with pClamp 10.2 software (Molecular Devices). For hM3Dq-expressing GnRH neurons, 1 μM CNO was added in the aCSF bathing medium using the perfusion system after stable baseline recording. Recordings were analysed using Clampfit 10.2 pClamp software (Molecular Devices). For each recording, the membrane potential and mean firing rate were determined before and during the bath application of drugs. Neurons were considered responsive if there was a >20% change in firing rate during CNO activation. Only cells that showed less than 20% change in access resistance throughout the recording were included in this study. The junction potential was corrected in the data analysis.

### iDISCO+: whole-mount ovarian immunolabeling

Ovaries were dissected following tissue perfusion, postfixed in 4% PFA overnight at 4 °C, and stored in 70% ethanol at 4 °C until further processing. We used and adapted an iDISCO+ protocol from Renier and colleagues^43^ to be used with ethanolic solutions. Samples were gradually dehydrated by washes in increasing concentrations of ethanol in 0.01 M PBS (70%, 75%, 80%, 90%, and twice 100% ethanol, 1 h for each step) under gentle rotation and at room temperature (RT). Samples were incubated in a solution of 66% dichloromethane (DCM; Sigma-Aldrich; #270997)/33% ethanol (VWR; #20821.310) under agitation at 4 °C overnight. Next, samples were washed twice in 100% ethanol at RT and bleached with 5% H_2_O_2_ (Fisher BioReagents; #BP2633-500) under agitation at 4 °C overnight. Samples were rehydrated in a decreasing concentration of ethanol in 0.01 M PBS (100%, 80%, 60%, 40%, 20%, and twice in PBS, 1 h for each step) under gentle rotation at RT. Next, samples were incubated in a blocking solution of 0.01 M PBS containing 0.2% gelatine (Fisher Scientific; #10075660), 1% Triton X-100 (Sigma; #9036-19-5), and 0.05% sodium azide (Fisher Scientific; #10592211) for preservation (PBS-GT) under rotation in an incubator at 37 °C for four days. Samples were transferred to a PBS-GT solution (2 mL/sample) containing the primary antibodies polyclonal rabbit anti-laminin (1:3000 dilution; Abcam, #ab11575) and monoclonal mouse anti-AMH (1:500 dilution; Abcam, #ab24542), and placed under rotation at 37 °C for two weeks. This was followed by six washes in PBS-GT for 1 h 30 min each at RT. Next, samples were incubated in PBS-GT with the secondary antibodies Alexa Fluor 568 donkey anti-rabbit IgG (Invitrogen; #A10042) and Alexa Fluor 647 donkey anti-mouse IgG (Invitrogen; A-31571), both at 1:500 dilution, under rotation at 37 °C for five days. This was followed by three washes in PBS-GT for 1 h 30 min each at RT. For easier handling a mounting, samples were included in 2% agarose gel providing a firm matrix and then dehydrated in increasing concentrations of ethanol in 0.01 M PBS (50%, 60%, 70%, 80%, 90%, and twice 100% ethanol, 45 min for each step) at RT. Next, samples were incubated with 66% DCM/33% ethanol under agitation at 4 °C overnight followed by two 45-min washes with 100% DCM under agitation at 4 °C. Clearing step was carried out using incubation in benzyl ether (Sigma-Aldrich, #108014) under agitation at RT for 2 h or until sample transparency was achieved.

### iDISCO+: ovarian 3D imaging and analysis

3D Imaging was performed as previously described^44^ on the Ultramicroscope I (LaVision BioTec) equipped with a 1.1×/0.1 NA and 4×/0.3 NA objectives and an Andor Neo 5.5 sCMOS camera. The light sheet was generated by a laser (wavelength 488 nm, 568 nm, or 647 nm, Coherent Sapphire Laser, LaVision BioTec) and two cylindrical lenses. Samples were placed in an imaging reservoir made of 100% quartz (LaVision BioTec) filled with DBE and illuminated from the side by the laser light. ImSpectorPro software (LaVision BioTec) was used for image acquisition, and the z-step between each image was fixed at 4 μm. The resulting sequences of tiff files were processed with Imaris Converter (Oxford Instruments) before visualization and analysis in Imaris 9.8 (Oxford Instruments). Counting and classification of the ovarian follicles were performed using the Imaris Spots tool: Four classes of spots were defined based on the morphology and shape of the follicles as previously established^27^^,^^45^: pre-antral, antral, and pre-ovulatory follicles and corpora lutea. The identity of each follicular type considered the presence (antral) or presence (pre-antral) of the antrum, the identification large size of a follicular space with antrum, cumulus oophorus, and corona radiata surrounding the oocyte (pre-ovulatory), and the presence of an agglomerated group of luteinized cells with average diameter of 480–550 μm (corpus luteum). Using both the 3D view and the 2D-slicer tool for confirmation, the ovaries were explored, and each follicle was assigned to the corresponding Spots class. For downstream analysis, the data from all four classes were exported using the statistics tab of the Spots tool. In addition, and to investigate potential differences in AMH signal intensity between groups at the organ level, the Imaris Surface tool and the 2D-slicer were used to manually segment the ovaries. The resulting surfaces allowed extraction of fluorescent signal information, including minimum, maximum, and mean voxel intensity, through the statistics tab of the tool for subsequent analysis.

### Organisms, reagents, antibodies references, and validation

All the detailed methods data regarding the source of organisms, reagents, antibodies, and tools’ references and their reference or validation are presented in the checklist table in Supplementary Table S1.

### Ethics statement

Animal studies were approved by the Institutional Ethics Committees for the Care and Use of Experimental Animals of the University of Lille and the French Ministry of National Education, Higher Education and Research (APAFIS#2617-2015110517317420 v5 and APAFIS#13387-2017122712209790 v9). All experiments were performed in accordance with the guidelines for animal use specified by the European Union Council Directive of September 22, 2010 (2010/63/EU). Animal studies were conducted in accordance with the ARRIVE (Animal Research: Reporting of *in vivo* Experiments) guidelines. All efforts were made to minimize animal suffering and animal care was supervised by veterinarians and animal technicians skilled in rodent healthcare and housing.

### Data analysis and statistics

All statistical analyses were performed using Prism 10.0.2 software (GraphPad Software, Dotmatics; San Diego, CA, USA). Normality was assessed using Q–Q plots followed by the confirmation from the Shapiro–Wilk test. The homogeneity of variance for hormone levels was assessed using Levene’s test and computed using Excel software combined with Prism 10.0.2 software. If the *P*-value of the Levene test was >0.05, then the variances were not statistically different from each other (the homogeneity assumption of the variance was met). Analysis from electrophysiological recordings used the Wilcoxon matched-pairs signed rank test as a non-parametric test to evaluate neuronal response before and after 1 μM CNO application. Repeated measures two-way ANOVA evaluated mostly two pairs of factors: time and type of treatment or time and phenotype. CNO-mediated dose-response curves were evaluated using repeated measures two-way ANOVA followed by Sidak’s multiple comparison test, and the area under the curve (AUC) analysis considered the total area summed over the 72-min blood sampling protocol. Estrous cycle analysis used a two-way ANOVA followed by Sidak’s multiple comparisons. Mann–Whitney *U* tests were used for all ovarian follicle count examinations. Analysis of testosterone levels used repeated measures two-way ANOVA with a post hoc Sidak’s test, while analysis of AMH levels used Kruskal–Wallis’ test with post hoc Dunn’s test. The sphericity assumption underlying the repeated measures ANOVA was always assessed and met in this study. Analysis of GnRH signaling blockade with Cetrorelix experiments used a two-way ANOVA followed by Sidak’s post hoc test, while correlation analysis used Spearman’s correlation method with an *r* coefficient between ±0.7 and 1 indicating a strong relationship between the two variables computed by Prism with a 95% of confidence interval (CI). Statistically significant *P* values were considered when *P* < 0.05. No statistical methods were used to pre-determine sample size, or to randomize. However, analyses were performed by two independent investigators in a blinded fashion and animals were randomly assigned to groups at weaning to minimize any potential bias. Animals were housed in same-sex groups of five animals per cage at weaning, with litters within the same treatment group mixed together (pseudo-random mixing based on weight). All written and graphical values are expressed as mean ± standard deviation (SD). Statistical differences are reported using either asterisk (∗) or hashtag (#) symbols for graphical representation. The number of the animals used for each experiment, together with the details of the statistical tests used for the analyses, are indicated in the figure legends of the corresponding figures.

### Role of funders

The funding sources for this project played no role in the study design, data collection, analysis, interpretation, writing, or editing of the manuscript.

## Results

### Selective chemogenetic targeting of hypophysiotropic GnRH neurons allows efficient control of GnRH neuronal activity and LH secretion

**Fig. 1:**
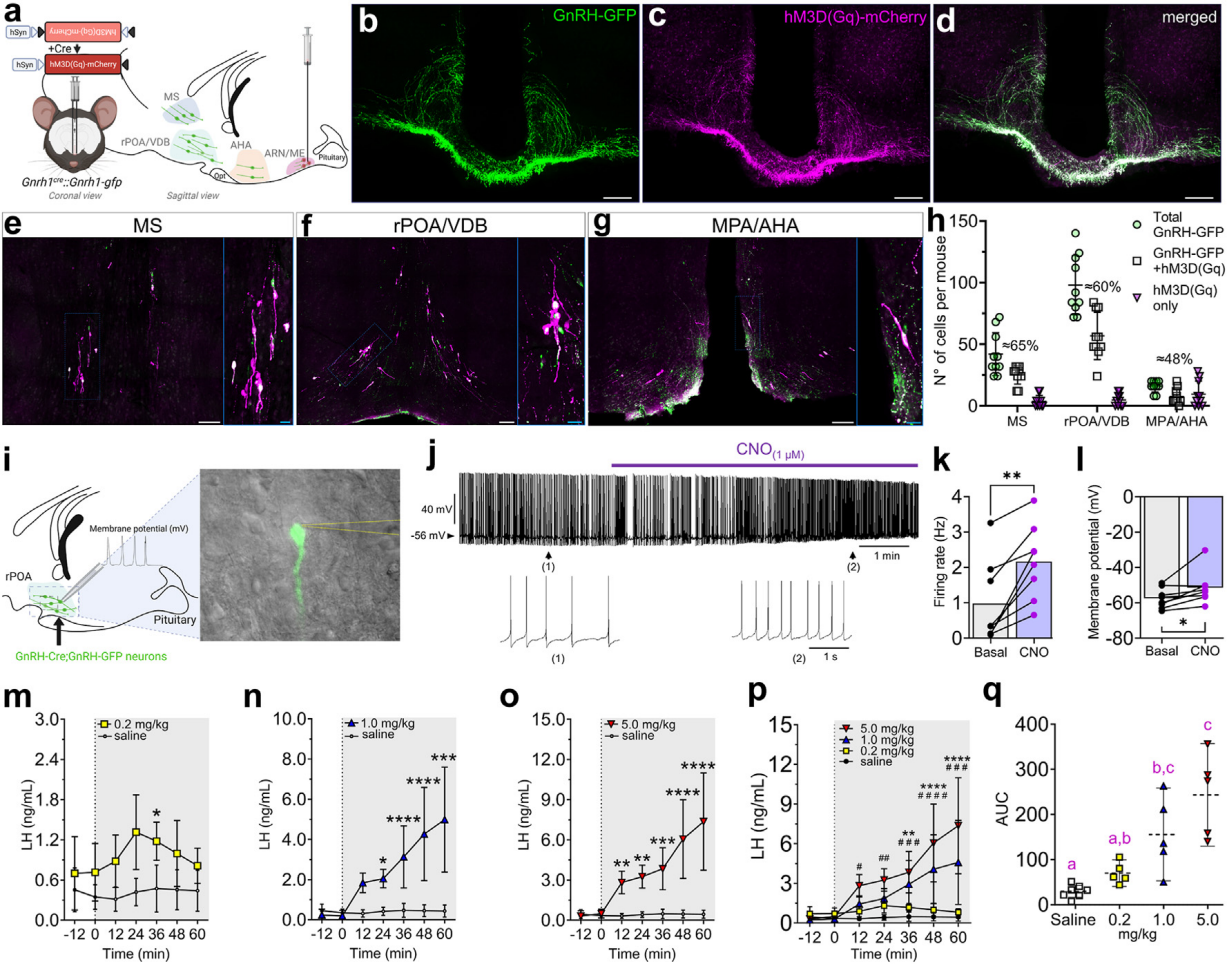
**Selective viral targeting enables chemogenetic activation of hypophysiotropic GnRH neurons.** (**a**) Illustration of the selective viral targeting for the expression of AAV9-hSyn-DIO-hM3D(Gq)-mCherry in hypophysiotropic GnRH neurons from adult *Gnrh1*^*cre*^*;Gnrh1-gfp* female mice. (**b–d**) Expression of GFP (green) and Cre-dependent mCherry (magenta), and colocalization following viral target into GnRH neurons at the level of the median eminence (ME) of the hypothalamus. Scale bar = 100 μm. (**e–g**) Expression and colocalization of GFP and AAV9-hSyn-DIO-hM3D(Gq)-mCherry in GnRH neurons in the medial septum (MS), rostral preoptic area/vertical diagonal band of Broca (rPOA/VDB), and medial preoptic area/anterior hypothalamic area (MPA/AHA). White scale bar = 100 μm; blue scale bar = 20 μm. (**h**) Graph shows the total number of GFP-expressing cells only (green circles), mCherry-expressing cells only (magenta triangles), and colocalization of both fluorophores (white squares) in four series of brain coronal slices in the MS, rPOA/VDB, and MPA/AHA (N = 10 mice). The approximate percentage of GFP and mCherry colocalization is shown at the top of its distribution. (**i**) Scheme shows in sagittal view the position of 200 μm coronal slices containing the rostral preoptic area (rPOA) where GnRH-GFP neurons were identified to perform whole-cell patch-clamp recordings. The expanded inset shows how GnRH-GFP neurons were visualized using an upright fluorescence microscope with infrared differential interference contrast and the recording pipette (dashed yellow lines). (**j**) Whole-cell patch-clamp voltage recordings from GnRH-GFP neurons expressing hM3D(Gq)-mCherry. The purple bar indicates the time of 1 μM CNO administration to the bath. Magnification insets show baseline (1) and post-CNO (2) recordings. (**k** and **l**) Histograms show average GnRH neuronal firing rate (**k**) and membrane potential (**l**) before and after 1 μM CNO administration to the bath. N = 6 mice; n = 8 cells; ∗*P* < 0.05, ∗∗*P* < 0.01; two-tailed Wilcoxon matched-pairs signed-rank test. (**m–p**) Chemogenetic activation of GnRH neurons evokes a dose-dependent change in luteinizing hormone (LH) secretion in female mice. Mice were treated either with saline-vehicle, 0.2 (**m**), 1.0 (**n**), or 5.0 (**o**) mg/kg of CNO (i.p.); statistical differences depicted comparisons between each CNO treatment and saline LH levels. Group analysis (**p**) shows CNO-evoked changes in LH secretion with comparisons within each group and their baseline LH levels. We detected statistical differences between the 1 mg/kg CNO and saline treatment (∗), and the 5 mg/kg CNO and saline treatment (#) over time. N_saline_ = 7; N_CNO doses_ = 5; repeated measures two-way ANOVA with Sidak’s test. (**q**) Integrated LH response using area under the curve (AUC) analysis from saline and CNO groups. N_saline_ = 7; N_CNO doses_ = 5; different letters indicate statistical differences among treatments. Kruskal–Wallis’ test with Dunn’s post hoc test. Data is shown as mean ± SD.

To establish an *in vivo* model of the overactivation of GnRH neurons in mice, we used the designer receptors exclusively activated by designer drugs (DREADD)-based chemogenetic tool hM3D(Gq). Transgenic mice expressing the green fluorescent protein (GFP) under the control of the GnRH promoter (*Gnrh1-gfp*),^46^ which allows optimal visualization of GnRH neurons, were crossed with heterozygous *Gnrh1*^*cre*^ mice^47^ to generate *Gnrh1*^*cre*^;*Gnrh1-gfp* female mice. Thus, we aimed to conditionally express the Cre-dependent virus AAV9-hSyn-DIO-hM3D(Gq)-mCherry in hypophysiotropic GnRH neurons from adult *Gnrh1*^*cre*^*;Gnrh1-gfp* female mice (12–16 weeks old). We selectively targeted hypophysiotropic GnRH neurons by the stereotaxic injection of the virus into the median eminence (ME), which is their termination field (Fig. 1a). The immunohistochemical evaluation showed that GnRH neuronal fibers were efficiently transfected at the level of the ME (Fig. 1b–d) and that viral retrograde transport allowed the detection of transfected cell bodies within the main locations of hypophysiotropic GnRH neurons: medial septum (MS), rostral preoptic area/vertical diagonal band of Broca (rPOA/VDB), and medial preoptic area/anterior hypothalamic area (MPA/AHA) (Fig. 1e–g). This approach yielded an effective viral expression of 64.8 ± 23.5%, 59.8 ± 22.8%, and 48.5 ± 34.6% in GnRH neurons located in the MS, rPOA/VDB, and MPA/AHA, respectively (N = 10; Fig. 1h). A small number of GnRH^MS^ neurons (12.6 ± 14.3%) and GnRH^rPOA/VDB^ neurons (7.0 ± 6.7%) expressed only hM3D(Gq)-mCherry (Fig. 1h). However, these numbers were higher for GnRH^MPA/AHA^ neurons (48.0 ± 36.2%), suggesting that GnRH promoter activity might be weak in the adult MPA/AHA.^48^

Clozapine-N-oxide (CNO) binding onto the activator DREADD hM3D(Gq) typically drives robust GnRH neuronal activation within seconds of its administration *in vitro*.^49^ To validate the efficacy of CNO to activate hypophysiotropic GnRH neurons, brain coronal slices were prepared for *in vitro* electrophysiological recordings of GnRH^rPOA/VDB^ neurons from *Gnrh1*^*cre*^*;Gnrh1-gfp* female mice stereotaxically injected with AAV9-hSyn-DIO-hM3D(Gq)-mCherry into the ME (Fig. 1i). Whole-cell path-clamp recordings revealed that the administration of 1 μM CNO robustly increased GnRH neuronal firing rate from an average baseline of 0.98 ± 1.17 Hz to CNO-stimulated values of 2.17 ± 1.05 Hz (Fig. 1j and k; N = 6 mice, n = 8 cells; *P* = 0.0078). We also observed that the average membrane potential following 1 μM CNO administration increased from −57.45 ± 5.74 mV to −51.47 ± 9.36 mV (Fig. 1l) indicating neuronal depolarisation.

Next, we sought to define the dose-response profile of the GnRH/LH secretion following *in vivo* CNO-mediated activation of hypophysiotropic GnRH neurons in diestrous female mice. *Gnrh1*^*cre*^*;Gnrh1-gfp* female mice stereotaxically injected with AAV9-hSyn-DIO-hM3D(Gq)-mCherry were acutely injected with either saline or three different doses of CNO (0.2, 1.0, and 5.0 mg/kg) coupled with tail-tip blood sampling to assess LH secretion. We found that these three doses were able to increase LH secretion in a dose-dependent manner with different hormone release dynamics. The 0.2 mg/kg CNO dose promoted a smaller increment in LH release 36 min after drug administration with fast recovery to baseline levels within the hour (Fig. 1m). Both 1.0 mg/kg and 5 mg/kg CNO doses promoted robust LH increase at 12 min of the drug administration with higher hormonal release increments reaching peak LH levels of ≈4.9 ng/mL and 7.4 ng/mL, respectively (Fig. 1n and o). Comparison group analysis indicated that only 1.0 mg/kg and 5 mg/kg CNO doses could promote a steady increase of LH secretion over time *in vivo* (Fig. 1p). We performed a further analysis of the integrated LH response based on previous studies^13^ and found statistical evidence that both 1.0 mg/kg and 5.0 mg/kg CNO doses enhance LH secretion suggesting that the latter might have reached a physiological ceiling threshold (Fig. 1q). Thus, the 1.0 mg/kg CNO was chosen for the following experiments to achieve optimal overactivation of GnRH neurons.

### Overactivation of GnRH neurons promotes long-term hyperandrogenism and disruption of reproductive cycles

Hyperandrogenism is highly frequent in women with PCOS, and it represents one of Rotterdam’s diagnostic traits. Thus, we first aimed to determine whether chronic overactivation of GnRH neurons is sufficient to induce a long-term elevation in circulating testosterone (T) levels in female mice.

**Fig. 2:**
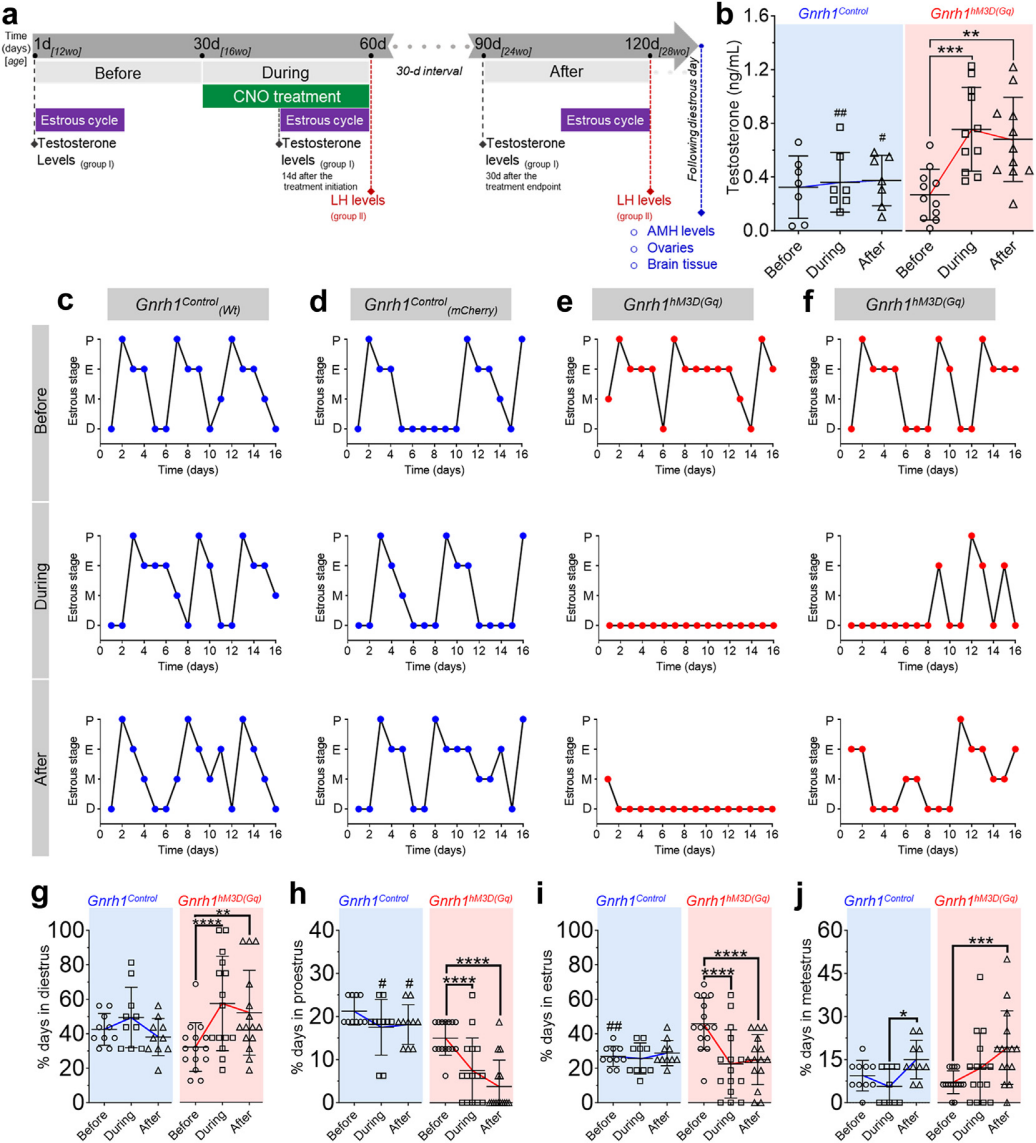
**Chronic overactivation of GnRH neurons promotes hyperandrogenism and disrupts reproductive cycles in female mice.** (**a**) Experimental timeline following a one-month chemogenetic activation protocol with 1 mg/kg CNO. The protocol had three different phases: *before*, *during*, and *after* the CNO regimen. Mouse age and treatment day for each phase are shown in the dark and light grey bars, respectively. Estrous cycles (purple bars) were followed for 16 consecutive days within each phase through vaginal smears evaluation. Between the *during* and *after* phases, all animals were given 30 days (dotted grey line). Testosterone and LH levels were measured from mouse groups I and II, respectively, and as indicated in the illustration. Blood sampling was performed on the day that the mice were in diestrus for both measurements. At the end of the protocol, we collected blood for AMH measurements, ovaries, and brains to validate proper stereotaxic injection sites and viral transfection. (**b**) Testosterone plasma levels *before*, *during*, and *after* the CNO regimen. N_control_ = 7; N_hM3D(Gq)_ = 11; repeated measures two-way ANOVA with Sidak’s post hoc test. (**c–f**) Estrous cycle profiles from *Gnrh1*^Control^, which were wild-type control (**c**) and mCherry control (**d**), and *Gnrh1*^hM3D(Gq)^ mice (**e** and **f**) at the *before*, *during*, and *after* phases of the CNO regimen. P = proestrus; E = estrus; M = metestrus; D = diestrus. (**g–j**) Analysis of the percentage of the time spent in each estrous cycle stage in *Gnrh1*^Control^ and *Gnrh1*^hM3D(Gq)^ female mice. The estrous cycle stage’s name is displayed on the y-axis. Statistical analysis shows differences within each mouse group (∗) among *before*, *during*, and *after* phases, and differences between groups (#) at each phase of the protocol. N_control_ = 10; N_hM3D(Gq)_ = 15; repeated measures two-way ANOVA with Sidak’s post hoc test. Data is shown as mean ± SD.

Adult female mice (16 weeks old) underwent a one-month treatment protocol with 1 mg/kg CNO, injected intraperitoneally (i.p.) every 48 h. The AAV9-hSyn-DIO-hM3D(Gq)-mCherry virus was injected into the ME of *Gnrh1*^*cre*^*;Gnrh1*^*gfp*^, hereafter referred to as *Gnrh1*^hM3D(Gq)^ mice (N = 11), The control group was composed of both C57BL/6 wild-type mice stereotaxically injected with AAV9-hSyn-DIO-hM3D(Gq)-mCherry (N = 4) and *Gnrh1*^*cre*^*;Gnrh1*^*gfp*^ female mice stereotaxically injected with control virus AAV9-hSyn-DIO-mCherry (N = 3), hereafter referred to as *Gnrh1*^Control^ mice (N = 7). Starting 4 weeks after intracranial viral injection, blood plasma samples were collected before, during the 1-month CNO treatment, and one or two months after its cessation (Fig. 2a). Before treatment initiation, T levels were similar between the two groups. However, T levels increased in *Gnrh1*^hM3D(Gq)^ mice 14 days after the start of the CNO treatment (*P* = 0.002) and remained high up to one month after treatment termination (*P* = 0.0014) (Fig. 2b). Specifically, T levels were ≈2-fold higher in *Gnrh1*^hM3D(Gq)^ mice than in *Gnrh1*^Control^ females. These results are compatible with our hypothesis that direct overactivation of GnRH neurons *in vivo* is sufficient to promote hyperandrogenism in adult females, recapitulating one of the hallmarks of PCOS.

Menstrual irregularities, another PCOS diagnostic trait, are highly prevalent in PCOS women^50^ and might be a consequence of androgen excess.^2^^,^^51^ It is also recognized that the combination of hyperandrogenism and irregular cycles during adolescence substantially increases the risk of PCOS during adulthood.^52^^,^^53^ Considering the importance of the detection of both diagnostic features of the disease, we evaluated the estrous cycle before, during, and after the chemogenetic overactivation protocol (Fig. 2a). Estrous cyclicity was either suppressed or markedly disrupted in *Gnrh1*^hM3D(Gq)^ mice and, intriguingly, remained altered after the cessation of the CNO treatment (Fig. 2c–j).

Together, these results uncovered that chemogenetic overactivation of GnRH neurons is likely to be sufficient to promote long-lasting disruption of reproductive cycles associated with androgen excess, closely resembling the PCOS condition.

### Overactivation of GnRH neurons sets the stage for PCOS-like ovarian dysfunction

**Fig. 3:**
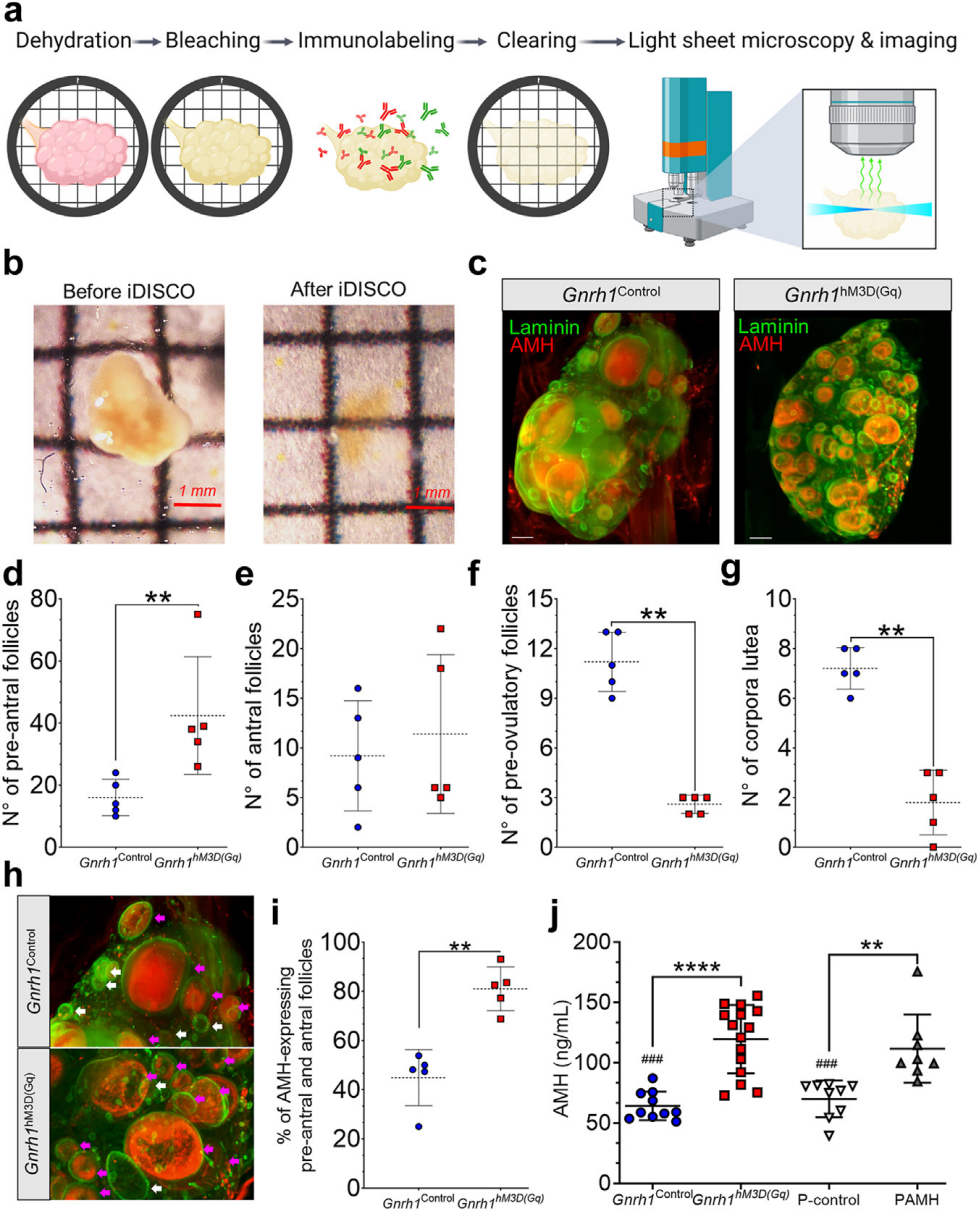
**Chronic overactivation of GnRH neurons triggers PCOS-like ovarian dysfunction in female mice.** (**a**) Illustration shows whole-mount ovarian immunolabeling using iDISCO+ pipeline, from tissue preparation through immunostaining and imaging using light sheet microscopy. (**b**) Level of optical transparency achieved in murine ovaries using iDISCO+ protocol. (**c**) Optical transparent ovaries from *Gnrh1*^Control^ and *Gnrh1*^hM3D(Gq)^ female mice showing laminin (green) and AMH expression. Scale bar = 300 μm. (**d–g**) Graphs show the number of pre-antral (**d**), antral (**e**), and pre-ovulatory follicles (**f**), and corpora lutea (**g**) following quantification in ovaries from *Gnrh1*^Control^ and *Gnrh1*^hM3D(Gq)^ female mice. N_control_ = 5; N_hM3D(Gq)_ = 5; Mann–Whitney *U* test. (**h**) High magnification of ovarian sections from images (**c**) showing in more detail AMH-expressing follicles (magenta arrows) and non-AMH-expressing follicles (white arrows). Scale bar = 300 μm. (**i**) Percentage of AMH-expressing pre-antral and antral ovarian follicles in ovaries from *Gnrh1*^Control^ and *Gnrh1*^hM3D(Gq)^ female mice. N_control_ = 5; N_hM3D(Gq)_ = 5; Mann–Whitney *U* test. (**j**) Plasma concentration of AMH in *Gnrh1*^Control^ (N = 10), *Gnrh1*^hM3D(Gq)^ (N = 15), prenatal control (*P*-control; N = 9) and prenatal AMH (PAMH; N = 8) female mice. Statistical analysis shows differences between controls and their PCOS model pairs (∗) and controls compared to the PCOS model of the adjacent group (#); Kruskal–Wallis test with Dunn’s post hoc test. Data is shown as mean ± SD.

Because we showed that overactivation of GnRH neurons during adult life induces oligo-anovulation in females, we speculated that GnRH hyperactivity might also induce long-term ovarian dysfunction. To test this hypothesis, we collected the ovaries and the blood from the animals two months after the CNO withdrawal (Fig. 2a). To assess the ovarian morphology, we capitalized on the iDISCO+ protocol^43^ to achieve *in toto* immunolabeling coupled with tissue clearing of the whole ovary and light-sheet microscopy (Fig. 3a and b). We performed immunolabeling for laminin, an extracellular matrix glycoprotein that labels the surface of ovarian follicular structures,^54^ and anti-Müllerian hormone (AMH), which participates in ovarian folliculogenesis and whose intraovarian and circulating levels are higher in women with PCOS as compared to normo-ovulatory women.^35^^,^^55^, ^56^, ^57^, ^58^ We observed that an ovarian morphological alteration was readily identified in *Gnrh1*^hM3D(Gq)^ female mice compared with *Gnrh1*^Control^ mice when looking at the gross and microscopic ovarian morphology (Fig. 3c; Supplementary Video S1). PCOS is often characterized by an altered ovarian morphology, with an arrest of antral follicular growth favouring the accumulation of pre-antral follicles and a decrease of follicular maturation,^27^^,^^59^, ^60^, ^61^ which results in the typical polycystic ovarian morphology of the syndrome at the ultrasound examination. Thus, we proceeded with a detailed morphometric analysis to evaluate the extent of ovarian dysfunction in our mouse model (Fig. 3c; Supplementary Video S2). Consistent with our hypothesis, quantitative analysis revealed an increase in the number of pre-antral follicles in *Gnrh1*^hM3D(Gq)^ female mice (*P* = 0.0079; Fig. 3d) whereas we did not detect any changes in the number of antral follicles (*P* = 0.85; Fig. 3e). In addition, we found a consistent reduction in the number of pre-ovulatory follicles (*P* = 0.0079; Fig. 3f) and corpora lutea (*P* = 0.0079; Fig. 3g), which is suggestive of impairment in ovulatory rates in these animals. Therefore, we hypothesized that a robust alteration of the ovarian follicular dynamics results from an overactivation of GnRH neurons in *Gnrh1*^hM3D(Gq)^ female mice recapitulating the ovarian abnormalities of the clinical condition.

Elevated AMH production is frequently observed in PCOS women during reproductive age^56^^,^^62^ and pregnancy,^35^^,^^57^^,^^63^ and it is considered to play an important role in the etiology of PCOS pathology and transmission of PCOS traits over multiple generations, through ovarian epigenetic changes.^64^ To assess whether ovarian dysfunction was associated with increased ovarian AMH expression in our mouse model, we first identified the percentage of both pre-antral and antral follicles that precisely expressed AMH as observed in the ovarian 3D data set (Fig. 3h). While 44.87 ± 11.39% of the pre-antral and antral follicles express AMH in *Gnrh1*^Control^ female mice, we found that these values were remarkably increased to 80.99 ± 8.91% in *Gnrh1*^hM3D(Gq)^ female mice (Fig. 3i). Serum AMH levels are positively associated with the antral follicular count in PCOS patients^65^; thus, we enquired whether elevated ovarian AMH expression in our *Gnrh1*^hM3D(Gq)^ female mice changes AMH levels in the circulation. We found that AMH levels in the blood were nearly 1.86-fold higher in *Gnrh1*^hM3D(Gq)^ female mice compared with controls (*P* < 0.0001; Fig. 3j) two months after CNO treatment was terminated. We further examined AMH levels from a well-established PCOS mouse model generated by prenatal AMH (PAMH) exposure^35^ to parallel our findings with this pre-clinical model. Results showed that PAMH mice present higher AMH levels compared to their prenatal control counterparts, injected with saline during late gestation (*P*-control) (*P* = 0.0068), but similar hormone levels to *Gnrh1*^hM3D(Gq)^ female mice (*P* = 0.79) (Fig. 3j). Across group pairs, we also observed that AMH levels were higher in both *Gnrh1*^hM3D(Gq)^ and PAMH mice compared with *P*-control and *Gnrh1*^Control^ females, respectively (Fig. 3j). Thus, our data suggest that central GnRH dysfunction may trigger persistent ovulatory impairments, by altering follicular development, intraovarian AMH expression, and circulating AMH levels, disturbances frequently observed in women with PCOS.

### Chemogenetic overactivation of GnRH neurons promotes long-term PCOS-like neuroendocrine dysfunction

**Fig. 4:**
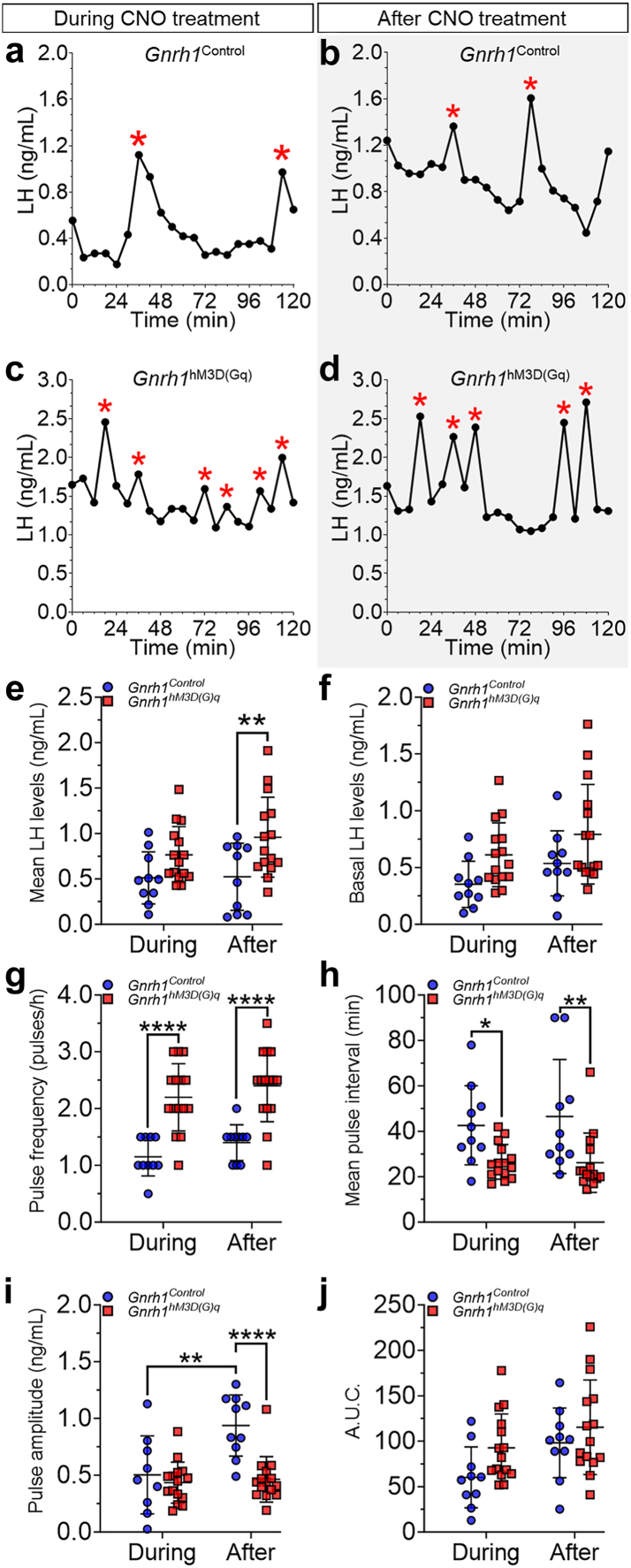
**Overactivation of GnRH neurons promotes long-term PCOS-like increase in luteinizing hormone (LH) secretion.** (**a–d**) Examples of LH pulse secretion profiles from the *during* (**a** and **c**) and *after* (**b** and **d**) protocol phases in *Gnrh1*^Control^ and *Gnrh1*^hM3D(Gq)^ female mice. Red asterisks depict LH pulses over the 2-h blood sampling period. (**e–j**) Group analysis of the LH pulse secretion in *Gnrh1*^Control^ and *Gnrh1*^hM3D(Gq)^ female mice. These graphs show the mean values of the LH levels (**e**), basal LH levels (**f**), LH pulse frequency (**g**), LH pulse interval (**h**), LH pulse amplitude (**i**), and integrated LH pulse response using the area under the curve (AUC) analysis (**j**). N_control_ = 10; N_hM3D(Gq)_ = 15; repeated measures two-way ANOVA with Fisher’s LSD post hoc test. Data is shown as mean ± SD.

The presence of PCOS-like cardinal features after the cessation of CNO treatment is likely to reflect persistent neuroendocrine disturbances in *Gnrh1*^hM3D(Gq)^ mice. Hence, we hypothesized that 4-week chemogenetic-induced changes in GnRH neurons are sufficient to entrench a long-term increase in LH pulse frequency such as in PCOS pathology. We aimed to assess LH levels during and after the chemogenetic overactivation protocol from tail-tip blood samples collected from diestrous/metestrous female mice over a 2-h bleeding session. As previously reported,^38^ the LH secretion profile in *Gnrh1*^Control^ female mice showed discrete episodes of one LH pulse per hour, on average, during and two months after our chronic regimen (Fig. 4a and b; N = 10). Conversely, we observed a clear increase in the detection of LH pulses over the same period in *Gnrh1*^hM3D(Gq)^ female mice (Fig. 4c and d; N = 15).

Analysis showed that, although LH levels seemed similar during treatment, mean LH levels were higher in *Gnrh1*^hM3D(Gq)^ compared with controls after the cessation of the CNO regimen compatible with our hypothesis (*Gnrh1*^Control^ = 0.52 ± 0.37 vs. *Gnrh1*^hM3D(Gq)^ = 0.96 ± 0.44 ng/mL; *P* = 0.0048; Fig. 4e). This elevation in mean LH levels does not seem to be followed by changes in basal LH levels for the evaluated time (*P* = 0.06; Fig. 4f). Evaluation of the LH pulse frequency revealed *Gnrh1*^hM3D(Gq)^ female mice displayed nearly twice as many pulses during (*Gnrh1*^Control^ = 1.15 ± 0.34 vs. *Gnrh1*^hM3D(Gq)^ = 2.20 ± 0.59 pulses/h; *P* < 0.0001) and >2 month after the cessation of the treatment (*Gnrh1*^Control^ = 1.40 ± 0.32 vs. *Gnrh1*^hM3D(Gq)^ = 2.40 ± 0.63 pulses/h; *P* < 0.0001) compared with *Gnrh1*^Control^ female mice (Fig. 4g). This increase in LH pulse frequency seems to be accompanied by a decrease in the mean LH pulse interval in *Gnrh1*^hM3D(Gq)^ females during (*Gnrh1*^Control^ = 42.60 ± 17.42 vs. *Gnrh1*^hM3D(Gq)^ = 26.51 ± 7.64 min; *P* = 0.017) and after treatment (*Gnrh1*^Control^ = 46.50 ± 25.11 vs. *Gnrh1*^hM3D(Gq)^ = 26.17 ± 13.04 min; *P* = 0.003) (Fig. 4h). The analysis also showed that the mean LH pulse amplitude between the groups was only be different after the treatment, in which *Gnrh1*^hM3D(Gq)^ female mice had shorter amplitudes of hormone secretion compared with *Gnrh1*^Control^ female mice (*Gnrh1*^Control^ = 0.94 ± 0.26 and *Gnrh1*^hM3D(Gq)^ = 0.46 ± 0.20 ng/mL; *P* < 0.0001; Fig. 4i). While the mean LH pulse amplitude remained unchanged for *Gnrh1*^hM3D(Gq)^ female mice during and after CNO treatment, the cessation of the regimen led to a compatible increase of this amplitude in *Gnrh1*^Control^ female mice comparing both time points (Fig. 4i). The integrated LH response to the totality of putative GnRH pulses was also calculated considering the area under the curve (AUC)^13^ for the total evaluated 2-h period and we did not detect statistical changes between the groups either during or after the CNO treatment (time vs. treatment: F_(1, 23)_ = 0.39; *P* = 0.53; Fig. 4j).

Together, these results demonstrate that a primary trigger of the overactivation of GnRH neurons might be sufficient to set an abnormally high pace of GnRH pulsatile secretion toward the promotion of long-lasting neuroendocrine dysfunction over reproductive life.

### PCOS-like neuroendocrine dysfunction and hyperandrogenemia are dependent upon exacerbated GnRH signaling

**Fig. 5:**
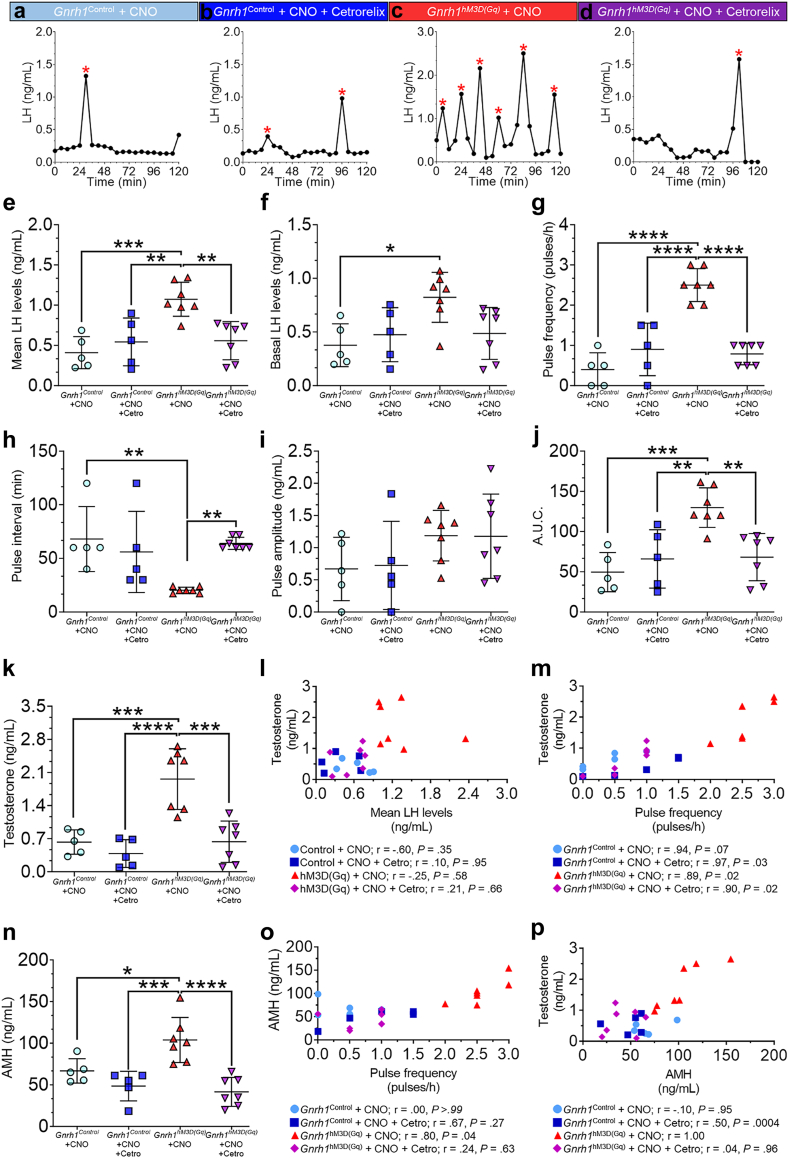
**GnRH signaling blockade precludes the establishment of long-term neuroendocrine dysfunctions and androgen excess in the hM3D(Gq) PCOS mouse model.** (**a–d**) Examples of LH pulse secretion profiles from *Gnrh1*^Control^ + CNO (**a**), *Gnrh1*^Control^ + CNO + Cetrorelix (**b**), *Gnrh1*^Control^ + CNO + Cetrorelix (**c**), and *Gnrh1*^hM3D(Gq)^ + CNO + Cetrorelix (**d**) groups. This protocol used a 1 mg/kg CNO regimen with concomitant treatment with s.c. 0.5 mg/kg Cetrorelix for one month and the hormonal assessment was performed two months after the chemogenetic intervention was finished. Red asterisks depict LH pulses over the 2-h blood sampling period. (**e–j**) Group analysis of the LH pulse secretion in *Gnrh1*^Control^ + CNO, *Gnrh1*^Control^ + CNO + Cetrorelix, *Gnrh1*^Control^ + CNO + Cetrorelix, and *Gnrh1*^Control^ + CNO + Cetrorelix groups. Graphs show the mean values of the LH levels (**e**), basal LH levels (**f**), LH pulse frequency (**g**), LH pulse interval (**h**), LH pulse amplitude (**i**), and integrated LH pulse response using the area under the curve (AUC) analysis (**j**). N_control + CNO_ = 5; N_control + CNO + Cetrorelix_ = 5; N_hM3D(Gq) + CNO_ = 7; N_hM3D(Gq) + CNO + Cetrorelix_ = 7; two-way ANOVA with Sidak’s post hoc test. (**k** and **n**) Plasma testosterone and AMH levels of the aforementioned groups following the CNO regimen and GnRH signaling blockade protocol. N_control + CNO_ = 5; N_control + CNO + Cetrorelix_ = 5; N_hM3D(Gq) + CNO_ = 7; N_hM3D(Gq) + CNO + Cetrorelix_ = 7; two-way ANOVA with Sidak’s post hoc test. (**l** and **m**) Correlation analysis between plasma testosterone levels with mean LH levels (**l**) and LH pulse frequency (**m**) using Spearman’s correlation coefficient (r) test. Correlation coefficients and statistical significance are shown under each graph. (**o** and **p**) Correlation analysis between plasma AMH levels with LH pulse frequency (**o**) and plasma testosterone levels (**p**) using Spearman’s correlation test. Correlation coefficients (r) and statistical significance (*P*) are shown under each graph.

To this point, our evidence suggested that central disturbances promoting high GnRH/LH secretion may robustly promote the common PCOS cardinal neuroendocrine and reproductive defects. Hence, we aimed to test whether these disruptive outcomes of GnRH neuron overactivation are mediated by the GnRH receptor (GnRH-R) and whether tempering GnRH-R signaling through a specific antagonist (Cetrorelix) may offer a prospective therapeutic strategy. Cetrorelix is an effective pharmacological tool, which saturates and down-regulates GnRH-R in gonadotropes of the pituitary gland suppressing LH levels.^66^^,^^67^ Our laboratory has shown that intermittent s.c. 0.5 mg/kg Cetrorelix treatment, given every 48 h normalizes LH levels and pulsatility, T levels, ovarian morphology, and estrous cycles in PCOS-like mice to control levels.^35^ This concentration was chosen as the one rectifying mean LH levels and LH pulsatility in PCOS-like mice, without blunting LH levels.^35^ Here, we performed the same chemogenetic GnRH neuron overactivation protocol with 1 mg/kg CNO as described above with concomitant treatment with Cetrorelix (s.c. 0.5 mg/kg/48 h) to evaluate their effects on hormone secretion in *Gnrh1*^Control^ (N = 5/group) and *Gnrh1*^hM3D(Gq)^ (N = 7/group) female mice two months after chemogenetic intervention. Importantly, this Cetrorelix treatment is shown to have no effects on endogenous LH secretory patterns in *Gnrh1*^Control^ mice (Fig. 5a and b).

While chemogenetic overactivation of GnRH neurons elevated LH pulse secretion *in vivo* (Fig. 5c), Cetrorelix co-administered with CNO prevented the aberrant elevation of LH pulse frequency in *Gnrh1*^hM3D(Gq)^ mice (Fig. 5d). Indeed, GnRH-R blockade may rescue normal mean LH levels in *Gnrh1*^hM3D(Gq)^ female mice (*Gnrh1*^hM3D(Gq)^ + CNO = 1.07 ± 0.21 vs. *Gnrh1*^hM3D(Gq)^ + CNO + Cetro = 0.56 ± 0.24 ng/mL; *P* = 0.0035) to circulating levels similar to control groups (*Gnrh1*^Control^ + CNO = 0.41 ± 0.19 and *Gnrh1*^Control^ + CNO + Cetro = 0.54 ± 0.29 ng/mL; *P* = 0.87 and *P* > 0.99, respectively) (Fig. 5e). Although we found a compatible increase in basal LH levels in *Gnrh1*^hM3D(Gq)^ + CNO mice compared with *Gnrh1*^Control^ + CNO mice (*Gnrh1*^hM3D(Gq)^ + CNO = 0.82 ± 0.23 vs. *Gnrh1*^Control^ + CNO = 0.37 ± 0.20 ng/mL; *P* = 0.023), we could not identify further statistical differences among the groups (Fig. 5f). We also found that GnRH-R blockade may prevent the establishment of high GnRH/LH pulse generation in *Gnrh1*^hM3D(Gq)^ mice following the rescue of both normal LH pulse secretion (*Gnrh1*^hM3D(Gq)^ + CNO = 2.50 ± 0.40 vs. *Gnrh1*^hM3D(Gq)^ + CNO + Cetro = 0.79 ± 0.27 pulses/h; *P* < 0.0001; Fig. 5g) and LH pulse interval (*Gnrh1*^hM3D(Gq)^ + CNO = 20.33 ± 2.82 vs. *Gnrh1*^hM3D(Gq)^ + CNO + Cetro = 64.00 ± 5.66 min; *P* = 0.0081; Fig. 5h). Although we did not detect any compatible differences in LH pulse amplitude among the groups (Fig. 5i), AUC analysis showed that Cetrorelix may prevent the total response of LH secretion to GnRH inputs in *Gnrh1*^hM3D(Gq)^ mice during the observed period (Fig. 5j; *Gnrh1*^hM3D(Gq)^ + CNO = 129.70 ± 24.62 vs. *Gnrh1*^hM3D(Gq)^ + CNO + Cetro = 68.17 ± 29.32 a.u.; *P* = 0.0035).

Following the LH secretion profile evaluation, we assessed plasma T and AMH levels from the same groups. Aligned with the aforementioned LH profile analysis, results showed that GnRH-R blockade with Cetrorelix may rescue normal plasma T levels in *Gnrh1*^hM3D(Gq)^ mice when compared to CNO treatment only (*Gnrh1*^hM3D(Gq)^ + CNO = 1.96 ± 0.64 vs. *Gnrh1*^hM3D(Gq)^ + CNO + Cetro = 0.63 ± 0.43 ng/mL; *P* < 0.0001) without altering T levels in *Gnrh1*^Control^ mice (*Gnrh1*^Control^ + CNO = 0.62 ± 0.25 and *Gnrh1*^Control^ + CNO + Cetro = 0.38 ± 0.29 ng/mL) (Fig. 5k). We further interrogated whether plasma T levels were associated with either mean LH levels or LH pulse frequency as these two factors are commonly reported in clinical and pre-clinical PCOS investigations when determining the hormonal consequence of neuroendocrine disturbances. Spearman’s correlation analysis showed that plasma T levels may not be correlated with mean LH levels in any of the four groups (Fig. 5l). Conversely, plasma T levels seem to be positively correlated with LH pulse frequency in all groups (*Gnrh1*^Control^ + CNO: r = 0.94; *Gnrh1*^Control^ + CNO + Cetro: r = 0.97; *Gnrh1*^hM3D(Gq)^ + CNO: r = 0.89; *Gnrh1*^hM3D(Gq)^ + CNO + Cetro: r = 0.90; Fig. 5m). We discovered that Cetrorelix treatment might be also effective to rescue normal AMH levels in *Gnrh1*^hM3D(Gq)^ mice when compared to CNO treatment only (*Gnrh1*^hM3D(Gq)^ + CNO = 103.90 ± 27.05 vs. *Gnrh1*^hM3D(Gq)^ + CNO + Cetro = 41.52 ± 17.38 ng/mL; *P* < 0.0001; Fig. 5n). Correlation analysis showed that AMH levels may only be positively correlated with LH pulse frequency (r = 0.80; Fig. 5o) and T levels (r = 1.00; Fig. 5p) in *Gnrh1*^hM3D(Gq)^ + CNO mice but not among the other groups.

These results show that exacerbated LH pulsatility, hyperandrogenism and increased AMH levels in PCOS may derive from enhanced GnRH signaling. As T and AMH excess seem more robustly correlated with high LH pulse frequency secretion, our results also suggest that both features might conjointly perpetuate the disordered brain-to-ovary communication in PCOS pathology.

## Discussion

High GnRH/LH secretion is a common pathophysiological trait in PCOS and is strongly associated with the disease’s diagnostic features.^10^^,^^13^^,^^16^^,^^18^^,^^19^^,^^68^ Despite all contemporary advances in reproductive health research, the causal link between abnormally high LH secretion and PCOS pathology has remained unsettled. Our study applied *in vivo* virogenetic manipulations to produce chronic overactivation of GnRH neurons recapitulating all PCOS-like cardinal features in female mice. Various mechanisms are likely involved in the pathophysiology of PCOS. However, in the present study, we demonstrate for the first time that the sole overactivation of GnRH neurons for a short lag of time in young adult mice is sufficient to entrain long-term deregulation of GnRH neuronal physiology cascading in a long-lasting state of neuroendocrine dysfunction, hyperandrogenism, and excessive AMH secretion. Our discoveries support the idea that a vicious cycle of neuro-hormonal imbalance might set the ground for reproductive impairments in PCOS spanning throughout the woman’s reproductive life.

A recent study using unsupervised clustering analysis using biochemical/hormonal and genotype data from more than 800 PCOS women could identify two main subtypes of PCOS: reproductive and metabolic.^69^ The PCOS reproductive subtype seems to be defined by high levels of LH and low BMI corresponding to nearly 25% of the evaluated PCOS population, and LH is one of the key traits distinguishing the reproductive subtype from the metabolic and indeterminate ones. In our study, we did not observe any changes in mouse body weight during the whole protocol (Supplementary Figure S1), and our model might be better suited when investigating the PCOS reproductive subtype. However, we did not follow up on these animals over the two months past the CNO withdrawal and we cannot exclude the possibility that metabolic abnormalities (i.e.: insulin resistance, dyslipidaemia, obesity) could manifest later in these animals. For example, in PAMH mice, metabolic features do not become apparent until 5–6 months of age^64^ and future investigations are required to address whether this is also the case for the preclinical virogenetic PCOS model that we generated in the current study. Here, we show that, by triggering a single frame of chronic GnRH neuronal overactivation, LH levels rise and remain elevated for at least two months, when we can detect substantial ovarian dysfunction and hyperandrogenism. Further analysis revealed that the frequency of LH secretion marks, and perhaps might be causative, of the described PCOS-like reproductive impairments. For instance, we found significant positive correlations between LH pulse frequency and either T or AMH levels. As the pulsatile nature of the GnRH/LH secretion is shared among different mammalian species,^21^^,^^22^ our results may support the assessment of the LH pulsatile profile as a potential predictive/diagnostic tool for PCOS in future clinical investigations. In addition, as far as we know, this is first report that shows that increased GnRH neuronal activity/secretion induces an elevation in circulating AMH levels, which is instead prevented upon GnRH antagonist treatment.

GnRH neurons are mostly found scattered along olfactory, septal, and hypothalamic areas with long projections reaching the median eminence of the hypothalamus where the GnRH peptide is secreted into the hypophyseal portal system.^22^^,^^70^ This unconventional distribution poses a great challenge when comes to targeting these cells with tools to manipulate and remotely control the activity of GnRH neurons *in vivo* while mechanistic studies using humans or non-human primates are mostly arduous with ethical and technical constraints. Chemogenetic manipulations of the entire GnRH neuronal population were achieved in a previous study using DREADDs technology with a focus on the putative role of an abnormal developmental switch of neuronal activity in PCOS.^49^ Here, we aimed to manipulate only hypophysiotropic GnRH neurons and, using this viral targeting approach, we identified nearly 60% of GnRH^MS^/GnRH^rPOA/VDB^ and 50% of GnRH^MPA/AHA^ neuroendocrine neurons in female mice. Bearing in mind the limitations of achieving full viral infection and surgical precision, we achieved similar results reported in studies elsewhere targeting the same neuronal population.^71^ Thus, this suggests that perturbations leading to an overexcitation of 50–60% of septal and hypothalamic GnRH neurons might be sufficient to generate neuroendocrine and reproductive impairments in females.

Increased GnRH/LH pulse secretion could be mostly mediated by reduced sensitivity of the GnRH pulse generation to the ovarian steroid-mediated negative feedback in PCOS. For instance, elevated T levels impair progesterone-mediated restrain of the GnRH secretion^68^^,^^72^ as PCOS patients are resistant to the suppressant effects of progesterone on GnRH/LH pulse frequency.^16^^,^^17^^,^^32^ Our results suggest that chronic androgen excess might be triggered by an initial central dysfunction of GnRH neurons while subsequent impairments (e.g.: decreased progesterone sensitivity within the GnRH neuronal network) are supported by androgen actions in the female brain.^73^, ^74^, ^75^, ^76^ Mammalian adult GnRH neurons do not express androgen receptor (AR), PR, and the canonical estrogen receptor alpha (ERα), which are critical for the proper function of the gonadal steroid hormone-mediated negative feedback.^77^, ^78^, ^79^ In addition, neuronal AR signaling is critical for the establishment of the disease’s full manifestation^80^, ^81^, ^82^ and for the decreased sensitivity to progesterone actions in PCOS mouse models.^76^ Our results showing that *Gnrh1*^hM3D(Gq)^ female mice present a lasting increase in LH pulse frequency after the termination of the CNO regimen suggest that androgen excess might impinge on key players of the GnRH pulse generation to maintain high LH release. Kisspeptin neurons located in the arcuate nucleus (ARN) of the hypothalamus (Kiss^ARN^) are considered to be the core of GnRH/LH pulse generation. Kiss^ARN^ neurons co-express kisspeptin, neurokinin B (NKB), dynorphin, and glutamate, which all play a part in setting the pace of the LH pulse release.^83^, ^84^, ^85^, ^86^ Studies using prenatally androgenized (PNA) rodent models report an increase in Kiss^75^^,^^87^ and NKB^87^, ^88^, ^89^ signaling and expression while dynorphin actions may be attenuated in PCOS.^90^ These molecular changes are also evinced in PCOS women^91^, ^92^, ^93^, ^94^ and are likely either programmed by prenatal androgen exposure or as the detrimental result of central androgen actions during adulthood. Our proposed model does not involve prenatal androgen insult, and yet alterations in LH pulse secretion are positively correlated with hyperandrogenism suggesting that adult androgen excess might be sufficient to alter the molecular frame within the GnRH pulse generator. Our study may pave the way for future research leveraging this new PCOS animal model to investigate androgen-dependent molecular and cellular changes in the GnRH pulse generator and its clinical implications in PCOS.

The induction of aberrant neuroendocrine activity in our model was able to yield substantial ovarian dysfunction recapitulating the human condition. Intra-ovarian hyperandrogenism is one culprit factor deregulating development in PCOS.^60^^,^^62^^,^^95^ Using a state-of-the-art approach with iDISCO+ technology and light sheet fluorescence microscopy, we show in detail that the overactivation of GnRH neurons promotes ovarian follicular arrest and robustly decreases the number of pre-ovulatory follicles and corpora lutea. Excessive ovarian androgen production is likely caused by an overstimulation of the gonad by LH onto theca cells where androgen synthesis takes place,^28^ while androgen actions also decrease granulosa cell proliferation^96^ and attenuate the progression of small ovarian follicles toward final maturation.^59^^,^^62^^,^^97^ Our study demonstrated that high GnRH neuron activity disrupts brain-ovarian communication via LH hypersecretion, which might trigger the initial step of follicular arrest in the PCOS-like condition. Along with this first trigger, the continuous ovarian dysfunction might be sustained by the increased intra-ovarian AMH production. In females, AMH is mostly produced by granulosa cells in the ovary of growing follicles working as a gatekeeper of the follicular development by inhibiting the transition of primordial to small pre-antral follicles and at a second transition point from small antral to preovulatory follicles.^58^^,^^62^ Hence, the identified high AMH levels in the blood two months after the end of the chemogenetic treatment could justify the abnormalities in ovarian follicle development of *Gnrh1*^hM3D(Gq)^ female mice. AMH is also considered a surrogate for PCOS-related hyperandrogenism and poses as a possible tool to diagnose PCOS.^65^^,^^98^ In addition, we have recently reported that PCOS women present enhanced neuronal activity and altered tuberal connectivity in the hypothalamus, which was associated with higher AMH levels compared with healthy women.^20^ We should also mention that AMH acts in the rodent’s brain and induces robust GnRH neuron activity and LH secretion.^55^ Herein, we also show that AMH is positively correlated with high LH pulse secretion and hyperandrogenism in a PCOS-like condition. Therefore, our model may provide additional insights into how AMH might contribute to PCOS pathophysiology through central and gonadal actions.

PCOS women of reproductive age typically present low to normal FSH levels^99^; however, due to high LH secretion, the LH:FSH ratio is commonly elevated in these patients compared to healthy women.^56^^,^^100^ Although we did not measure FSH nor estradiol levels in this study, we may speculate that FSH release and FSH-mediated actions in the ovaries might be decreased following chronic activation of GnRH neurons. Firstly, high GnRH frequency signaling via GnRH-R binding onto the pituitary gland is known to attenuate FSH synthesis while favouring LH release in mammals.^101^ Low FSH levels are also causative for low estradiol synthesis. Secondly, gonadal FSH-mediated actions might be attenuated due to high AMH levels as the latter is known to counteract FSH actions locally in ovarian granulosa cells and could indirectly attenuate estrogenic synthesis in females.^102^^,^^103^

We previously proposed, in a preclinical study, that reduced, but not blunted, LH secretion through partial GnRH-R antagonism may be a promising pharmacological tool to treat PCOS.^35^ In line with those findings, we discovered that the delivery of low doses of Cetrorelix concomitantly with the chemogenetic GnRH neuronal activation is sufficient to prevent the aberrant elevation of LH pulse frequency and hyperandrogenism in *Gnrh1*^hM3D(Gq)^ mice.

Besides its key role in the regulation of reproduction, we recently provided evidence that GnRH is also involved in a variety of non-reproductive functions, including chemosensory processing^44^ and those involved in intellectual functions.^104^ Indeed, we have previously reported that a portion of GnRH neurons extend projections to extra-hypothalamic cognitive areas expressing GnRH-R.^104^ Taking advantage of a trisomic mouse model of Down Syndrome (DS; Ts65Dn mice) which displays subfertility and progressive cognitive impairments similar to that of DS patients, we have highlighted that these nonreproductive neurological symptoms closely paralleled a postpubertal loss of GnRH neurons and fibers in the hypothalamus as well as in extrahypothalamic regions, which is reflected by changes in the levels and pattern of release of LH in the blood.^104^ Strikingly, pulsatile GnRH therapy improved cognition both in Ts65Dn mice and in DS patients, as shown in our pilot clinical trial,^104^ and pointed to optimal GnRH/LH pulsatility as being a necessary component for cognitive functions. As PCOS patients are at higher risk of developing anxiety, depression, sexual dysfunction, and cognitive dysfunction,^105^ our model could be used to access the central mechanisms behind these impairments in PCOS research.

Importantly, in this study, we have shown that GnRH-R antagonism has no effects on endogenous LH secretory pattern in *Gnrh1*^Control^ mice, whereas Cetrorelix co-administered with CNO prevented the aberrant elevation of LH pulse frequency in *Gnrh1*^hM3D(Gq)^ mice. These results suggest that GnRH-R antagonist treatment, when delivered at low doses that do not blunt LH secretion, is not harmful to cognition.

Some limitations should be considered. First, we are aware of the presence of few confounding factors as we did not measure baseline LH levels before Cetrorelix treatment, which could be influencing post-treatment LH, AMH, and T outcomes. In addition, although we showed that we can efficiently target and modulate 50–60% of septal and hypothalamic GnRH neurons using our Cre-dependent chemogenetic strategy, which is sufficient to drive PCOS-like neuroendocrine traits, we cannot rule out the possibility that downstream neuronal targets of GnRH neurons may be also activated upon the chemogenetic protocol. Indeed, we recently showed that hypophysiotropic GnRH neurons also project to extra-hypothalamic areas involved in cognitive processes^104^ and the possibility that chronic GnRH activation over a sustained lag of time may impact on these functions should be considered in future studies.

In conclusion, our discoveries demonstrate that the mere increase in GnRH neuron activity to pathological levels over a short period of time is sufficient for the establishment of long-term neuroendocrine dysfunction and androgen excess in PCOS-like conditions. Remarkably, the GnRH-R blockade precludes the establishment of long-term neuroendocrine dysfunction and androgen excess. Normalizing the exacerbated GnRH signaling could be thus an efficient therapeutic venue for the treatment of PCOS, likely by placing women on a long-term regimen GnRH antagonist treatment throughout their reproductive lives since the effects of Cetrorelix on LH secretion are reversible after discontinuation of the treatment. However, it remains to be assessed in future studies whether long-term preclinical treatments (i.e.: 3–6 months) with low doses of GnRH-R antagonists may have a potential benefit in correcting neuroendocrine and reproductive alterations of PCOS-like mice. Another interesting observation of this study is that androgen excess and abnormally high AMH levels are robustly correlated with high LH pulse secretion suggesting that both features might conjointly perpetuate the disordered brain-to-ovary communication in the PCOS pathology.

## Contributors

M.S.B.S. and P.G. designed the experimental plan; M.S.B.S., L.D., G.D., T.L., and G.T. performed research; P.G. and V.P. contributed with mice and analytic tools; M.S.B.S., L.D., G.D., T.L., G.T., and P.G. analysed data; M.S.B.S. and P.G. designed the study, analysed, and verified the underlying data and wrote the manuscript. All authors critically read and commented on the manuscript and approved the final version for submission.

## Data sharing statement

Study protocol and all data collected for the study, including raw data and data analysis will be made available to others upon request. All data will be available upon publication of the manuscript, by contacting the corresponding author.

## Declaration of interests

P.G. and V.P. disclose that they are inventors of a patent application by the INSERM (Institut National de la Santé et de la Recherche Médicale) covering the use of GnRH antagonists for the treatment of women affected with PCOS (N° of publication: WO2018177746). All other authors do not have competing interests.
