# Supplementary material for: Overactivation of GnRH neurons is sufficient to trigger polycystic ovary syndrome-like traits in female mice
Source: eBioMedicine. 2023 Oct 27;97:104850. doi: 10.1016/j.ebiom.2023.104850 (PMC10630624; doi:10.1016/j.ebiom.2023.104850)
Supplement: Supplementary Table S1 and Figure S1 [file mmc1.pdf]

Supplementary Information

Overactivation of GnRH neurons is sufficient to trigger polycystic ovary syndrome-like traits in female mice.

Mauro S. B. Silva[1,2,†], Laurine Decoster[1,2], Gaspard Delpouve[1,2], Tori Lhomme[1,2], Gaetan Ternier[1,2], Vincent Prevot[1,2], and Paolo Giacobini[1,2,*]

[1] Laboratory of Development and Plasticity of the Neuroendocrine Brain, FHU 1000 days for health, School of Medicine, Lille, France

[2] Univ. Lille, Inserm, CHU Lille, Lille Neuroscience & Cognition, UMR-S 1172, Lille, France

† New address: Department of Medicine, Division of Endocrinology, Diabetes and Hypertension, Brigham and Women's Hospital and Harvard Medical School, Boston, MA, USA.

* Correspondence to: paolo.giacobini@inserm.fr

Table of contents

Supplementary Table 1: Checklist of organisms, reagents, antibodies, and tools……………………..p. 2

Supplementary Figure S1……………………………………………………………………………p. 3

Supplementary Table 1: Checklist of organisms, reagents, antibodies, and tools.

Organism/ antibody/ reagent/ tool	Source	Catalogue number	RRID	GenPept/ PubChem/ Cas	Validation/ Reference
Gnrh1 ^{cre} mice	Donation from Dr. Dullac (*Harvard University*)	#:021207	IMSR_JAX:021207	NA	PubMed:16290037
C57BL/6J mice	Charles River, France	Strain Code: 632	IMSR_JAX:000664	NA	NA
recombinant Human MIS/AMH	R&D Systems	1737-MS-10	NA	P03971.3	PubMed: 36356855
Clozapine N-oxide dihydrochloride	Tocris	6329	NA	137187247	PubMed: 30523062
Cetrorelix acetate	Sigma-Aldrich	C5249	NA	329775083	PubMed: 2405399
paraformaldehyde	Thermo Scientific Chemicals	A11313.22	NA	Cas 30525-89-4	NA
Normal Donkey Serum (NDS)	Jackson ImmunoResearch Inc.	017-000-121	AB_2337258	NA	NA
chicken anti-GFP	Aves-Lab	GFP-1010	AB_2307313	NA	Pubmed: 36384114
rabbit anti-RFP	Rockland Immunochemicals	600-401-379	AB_2209751	NA	PubMed: 35108514
rabbit anti-laminin	Abcam	ab11575	AB_298179	NA	PubMed: 35076391
mouse anti-AMH	Abcam	ab24542	AB_2801539	NA	PubMed: 31291191
donkey anti-mouse Alexa Fluor 647	Invitrogen	A-31571	AB_162542	NA	PubMed: 37648867
donkey anti-chicken Alexa Fluor 488	Thermo Fisher Scientific	SA1-72000	AB_923386	NA	NA
donkey anti-rabbit Alexa Fluor 568	Invitrogen	A10042	AB_2534017	NA	NA
Fluoromount-GTM with DAPI	Invitrogen	00-4959-52	NA	NA	NA
dichloromethane	Sigma-Aldrich	270997	NA	57648216	NA
ethanol absolute	VWR; BDH Chemicals	20821.31	NA	Cas 64-17-5	NA
hydrogen peroxide (30%)	Fisher Scientific	BP2633500	NA	Cas 7722-84-1	NA
gelatine	Fisher scientific	10075660	NA	Cas 9000-70-8	NA
Triton X-100	Sigma-Aldrich	9036-19-5	NA	329830772	NA
sodium azide	Fisher scientific	10338380	NA	33557	NA
benzyl ether	Sigma-Aldrich	108014	NA	24846794	NA
Photoshop CS5	Adobe Systems	NA	NA	NA	NA
Imaris 9.8	Oxford Instruments	NA	SCR_007370	NA	NA
ImageJ Software	National Institutes of Health, Bethesda, MD	NA	SCR_003070	NA	PubMed: 22930834
Leica VT1200S vibratome	Leica Biosystems	NA	SCR_018453	NA	NA
Leica DM LFS microscope	Leica Microsystems	DM2500	NA	NA	NA
Ultramicroscope I	LaVision BioTec	NA	NA	NA	NA
Prism 10.0.2 software	GraphPad Software, Dotmatics	NA	SCR_002798	NA	NA

Supplementary Table 1. Organisms, reagents, antibodies references, and validation. The table shows the source, catalogue number, research resource identifier (RRID), protein database entry number (GenPept), chemical database entry number (PubChem), chemical abstracts service (Cas) registry number, and research papers containing either reference for use or source validation for all organisms, antibodies, main reagents, and tools used in this study. NA = not applicable.

Supplementary Figure S1

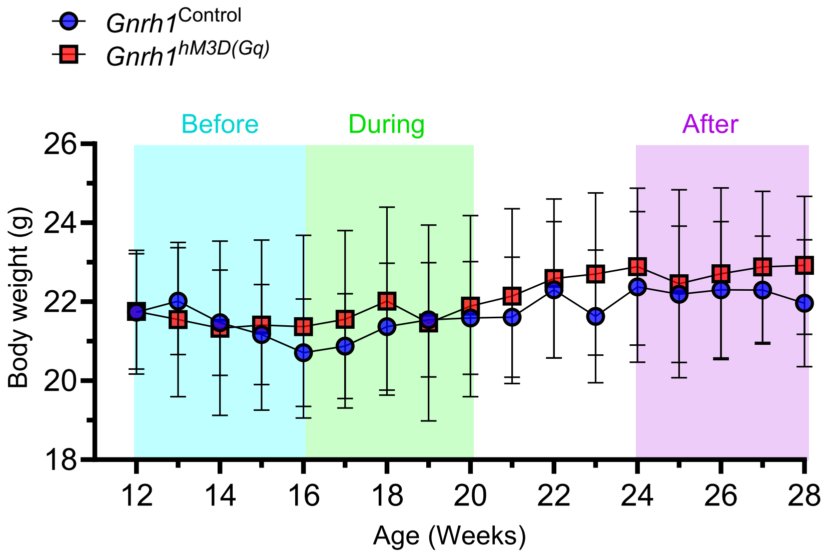

Supplementary Figure S1. Chronic chemogenetic activation of GnRH neurons does not alter body weight. Mouse body weight *(in grams*) was followed once every week from 4 weeks of age to 28 weeks of age for *Gnrh1*^{Control} and *Gnrh1*$^{\mathrm{hM3D(Gq)}}$ female mice. Coloured areas indicate before (*blue*), during (*green*), and after (*purple*) phases of the CNO regimen as represented in Figure 2. N_{control} = 10; $N_{\mathrm{hM3D(Gq)}}$ = 15; repeated measures two-way ANOVA with Sidak's post hoc test. Data is shown as mean ± SD.
